# Comparison of Autof ms1000 and Bruker Biotyper MALDI-TOF MS Platforms for Routine Identification of Clinical Microorganisms

**DOI:** 10.1155/2021/6667623

**Published:** 2021-03-03

**Authors:** Jae Hyeon Park, Yujin Jang, Inseon Yoon, Taek Soo Kim, Hyunwoong Park

**Affiliations:** ^1^Department of Laboratory Medicine, Seoul National University College of Medicine, 03080 Seoul, Republic of Korea; ^2^Department of Laboratory Medicine, Seoul National University Hospital, 03080 Seoul, Republic of Korea; ^3^Biomedical Research Institute, Seoul National University Hospital, 03080 Seoul, Republic of Korea; ^4^Department of Laboratory Medicine, Seoul National University Boramae Medical Center, 07061 Seoul, Republic of Korea

## Abstract

Matrix-assisted laser desorption ionization-time of flight mass spectrometry (MALDI-TOF MS) is widely used in clinical microbiology laboratories because it is cost-effective, reliable, and fast. This study is aimed at comparing the identification performance of the recently developed Autof ms1000 (Autobio, China) with that of the Bruker Biotyper (Bruker Daltonics, Germany). From January to June 2020, 205 preserved strains and 302 clinical isolates were used for comparison. Bacteria were tested with duplicates of the direct transfer method, and formic acid extraction was performed if the results were not at the species level. Fungi were tested with formic acid extraction followed by ethanol extraction methods. 16S rRNA or ITS region sequence analysis was performed on isolates that could not be identified by any of the instruments and on isolates that showed inconsistent results. The time to result of each instrument was also compared. Among preserved strains, species-level identification results were obtained in 202 (98.5%) strains by the Autof ms1000 and 200 (97.6%) strains by the Bruker Biotyper. Correct identification at the species/complex level was obtained for 200 (97.6%) strains by the Autof ms1000 and for 199 (97.1%) strains by the Bruker Biotyper. Among clinical isolates, species-level identification results were obtained in 301 (99.7%) strains and 300 (99.3%) strains by the Autof ms1000 and Bruker Biotyper, respectively. Correct identification at the species/complex level was achieved for 299 (99.0%) strains by the Autof ms1000 and for 300 (99.3%) strains by the Bruker Biotyper. The time to analyze 96 spots was approximately 14 min for the Autof ms1000 and approximately 27 min for the Bruker Biotyper. The two instruments showed comparable performance for the routine identification of clinical microorganisms. In addition, the Autof ms1000 has a short test time, making it convenient for use in clinical microbiology laboratories.

## 1. Introduction

The identification of microorganisms relies on phenotypic and biochemical characteristics. The process of identification has been automated but remains laborious and time consuming. Currently, molecular identification is the standard method, but it is not routinely used in clinical microbiology laboratories due to cost and labour requirements. Matrix-assisted laser desorption ionization-time of flight mass spectrometry (MALDI-TOF MS) has been rapidly introduced into laboratories because it is a rapid, economical, and accurate method for the routine identification of various microorganisms [1, 2]. The first developed Bruker Biotyper (Bruker Daltonik GmbH, Leipzig, Germany) and VITEK MS (bioMérieux, Marcy l'Etoile, France) received FDA and CE-marked IVD approval. Their identification performances in clinical microbiology laboratories have been evaluated in several countries [3–8]. The strengths and limitations of MALDI-TOF MS have also been reported [1, 2]. Therefore, many laboratories are changing their routine identification methods from biochemical methods to MALDI-TOF MS. Since these events, various MALDI-TOF MS instruments with similar principles have been developed. These new instruments, such as the MicroIDSys system (ASTA corp., Suwon, South Korea) [9–11], Microtyper MALDI-TOF MS (Xiamen Mass Spectrometry, Xiamen, China) [12], and Clin-TOF (Bioyong Technologies, Beijing, China) [13], have been proven to be comparable to conventional MALDI-TOF MS.

The Autof ms1000 (Autobio Diagnostics in Zhengzhou, China) is a MALDI-TOF MS instrument developed in April 2018 that received CE-marked IVD clearance in June 2018. It is characterized by the use of the latest cloud database and an external pump to achieve vacuum rapidly. The use of the Autof ms1000 has been reported in a case of *Trichosporon dohaense* infection [14], a novel isolate of *Legionella qingyii* [15], and an evaluation in *Bacteroides fragilis* group isolates [16]. Recently, the Autof ms1000 was evaluated for clinical isolates in China, and it showed comparable identification performance to the Bruker Biotyper [17]. Since it was tested on commonly isolated microorganisms, a comprehensive comparison was insufficient. In addition, the performance of the Autof ms1000 for routine identification in clinical microbiology laboratories outside China has not been evaluated.

This study is aimed at comparing the diagnostic performance of the Autof ms1000 with that of the Bruker Biotyper. The identification performance was evaluated in preserved strains and clinical isolates, and the analysis time was compared.

## 2. Materials and Methods

### 2.1. Evaluated Microorganisms

The evaluation was divided into the use of preserved strains for the overall performance on various species and of clinical isolates for performance in routine isolates. A total of 205 strains stored in the clinical microbiology laboratory of Seoul National University Hospital from 2015 to 2020 were used as preserved strains. They included strains isolated from clinical samples and American Type Culture Collection (ATCC) strains. Clinical isolates were cultured from routine specimens received from February 2020 to June 2020. A total of 302 clinical isolates was tested, including genitourinary, respiratory, blood, gastrointestinal, and other specimens. This study was approved by the institutional review board of Seoul National University Hospital (IRB No. 1911-109-1080).

### 2.2. MALDI-TOF MS Analysis

The frozen aerobic bacteria and yeast strains were subcultured twice on blood agar plates (BAPs) or on Sabouraud dextrose agar. The anaerobic bacteria were subcultured twice on Brucella agar in an anaerobic jar. After the culture, in parallel, the same medical technician took the colony from the same medium and placed it on the target plate. Bacteria and fungi were transferred to the target plate by the direct transfer method and the formic acid (FA) method. Initial analysis of each strain was performed in duplicate, and the results with a higher range/score were used. If the results of both instruments were not species-level identification, further tests were conducted. For further tests, FA extraction was used for bacteria, and ethanol/formic acid (EtOH/FA) extraction was performed for fungi.

### 2.3. Autobio Autof ms1000

All procedures were performed according to the manufacturer's instructions. The test was performed using Autobio Autof ms1000, and Acquirer 1.0.151 and local DB version 1.1.11 were used to analyze the results. The database has mass spectra of 14,174 strains and 4,226 species. The Autof ms1000 instrument was calibrated daily using the calibrator. Internal quality control was performed with *Staphylococcus aureus*, *Escherichia coli*, *Candida albicans*, and a blank spot. The target plate of Autof ms1000 is disposable and has 96 spots. In the direct transfer procedure, a single colony was smeared directly onto the target plate. After drying, 1 *μ*L of matrix solution (*α*-cyano-4-hydroxycinnamic acid, CHCA) was applied to the target plate and dried at room temperature. In the FA extraction method, a single colony was smeared onto the target plate, 1 *μ*L of 70% FA was applied and dried, and then the matrix solution was applied. The EtOH/FA extraction method was performed as follows. A single colony was mixed with 300 *μ*L of HPLC grade deionized water and 900 *μ*L of ethanol in a 1.5 mL Eppendorf tube. After vortexing, the Eppendorf tube was centrifuged for 2 min at 13,000 rpm, and the supernatant was removed. The pellet was air-dried and resuspended in 10 *μ*L of FA. After vortexing, 10 *μ*L of acetonitrile (ACN) was added, and the tube was vortexed and then centrifuged for 2 min at 13,000 rpm. One microlitre of the supernatant was then applied to the target plate and dried at room temperature. Then, 1 *μ*L of the matrix solution was applied to the target plate and dried at room temperature. Each result is listed in a ranking table of the identification results and range values. The identification result was interpreted according to the manufacturer's recommendations as “reliable species identification” for range 9.000-10.000, “reliable genus identification” for range 6.000-8.999, and “no reliable identification” for range < 6.000.

### 2.4. Bruker Biotyper

All procedures were performed according to the manufacturer's instructions. Mass spectrometry was performed using a Bruker microflex LT. The laser source has not yet been replaced. MALDI Biotyper RTC software 3.1 and 6903 MSP (main spectra) Library were used for analysis. A Bruker microflex LT instrument was calibrated weekly using the bacterial test standard (BTS, Bruker Daltonics). Internal quality control was performed with *S. aureus*, *E. coli*, *C. albicans*, and a blank spot. The procedures of Bruker Biotyper in the direct transfer method, FA extraction method, and EtOH/FA extraction method were similar to those of Autof ms1000. The difference was that the volume of FA and ACN was 50 *μ*L in EtOH/FA extraction. Each result is listed in a ranking table of the identification results and score values. The identification result was interpreted according to the manufacturer's recommendations as “reliable species identification” for score 2.00-3.00, “reliable genus identification” for score 1.70-1.99, and “no reliable identification” for score < 1.7.

### 2.5. Comparison of the Identification Results

The agreement of the results was compared at the species/complex level and the genus level. The species-level agreement was the same species-level result obtained by both MALDI-TOF MS instrument methods, and due to the limitations of MALDI-TOF MS, bacteria that were known to be difficult to identify accurately at the species level were compared at the complex level. *Acinetobacter baumannii* complex [18, 19] (*A. baumannii*, *A. pittii*, *A. nosocomialis*, *A. lactucae*, and *A. calcoaceticus*), *Citrobacter freundii* complex [20] (*C. freundii*, *C. koseri*, *C. braakii*, *C. youngae*, *C. werkmanii*, *C. sedlakii*, *C. amalonaticus*, and *C. farmeri*), *Enterobacter clocae* complex [21] (*E. cloacae*, *E. asburiae*, *E. hormaechei*, *E. kobei*, *E. ludwigii*, and *E. nimipressuralis*), *Klebsiella pneumoniae* complex [22] (*K. pneumoniae*, *K. quasipneumoniae*, and *K. variicola*), and *Burkholderia cepacia* complex [23] (*B. cepacia*, *B. cenocepacia*, *B. metallica*, etc.) were compared in complex results. The genus-level agreement was the same genus-level result obtained by both MALDI-TOF MS instruments.

### 2.6. Molecular Identification of Discrepant Results

If the two devices' results were both at the species level but did not match, sequence analyses were performed at Macrogen (Seoul, South Korea). Bacteria were subjected to 16S rRNA sequencing and fungi to internal transcription spacer (ITS) region sequencing. The primers used to amplify the 16S rRNA gene of the isolate were 5′-AGAGTTTGATCMTGGCTCAG-3′ (27F) and 5′-TACGGYTACCTTGTTACGACTT-3′ (1492R), and the primers used for fungi were 5′-TCCGTAGGTGAACCTGCGG-3′ (ITS1) and 5′-TCCTCCGCTTATTGATATGC-3′ (ITS4). The sequences were compared with sequences in the GenBank database using BLAST software (https://blast.ncbi.nlm.nih.gov/Blast.cgi) and interpreted according to CLSI guidelines [24].

### 2.7. Comparison of the Time to Analyze

To compare the devices equally, a worklist was prepared in advance, and the time taken for the actual analysis was measured. After mounting the target plate to the device, the time to load a target plate and make a vacuum to prepare for starting the test and the time to analyze spots and report results were measured. The number of tested spots was divided into 12, reflecting actual use in laboratories and 96 in total.

### 2.8. Statistical Analysis

Descriptive variables are presented as frequencies, and continuous variables are presented as medians and interquartile ranges (IQRs). Groups were compared using the McNemar test for categorical characteristics and the Mann–Whitney test for continuous characteristics. *P* < 0.05 was considered statistically significant. Statistical analyses were conducted using R software version 4.0.0 (R Project for Statistical Computing, Vienna, Austria).

## 3. Results

### 3.1. Identification Results in Preserved Strains

Identification performance was evaluated in 205 preserved strains, including 193 clinical strains and 12 ATCC strains. They included 91 Gram-negatives, 80 Gram-positives, 12 anaerobes, and 22 fungi. The strains comprised 29 (14.1%) *Staphylococcus*, 23 (11.2%) *Streptococcus*, 22 (10.7%) *Candida*, 21 (10.2%) *Enterococcus*, 12 (5.8%) *Klebsiella*, 11 (5.3%) *Enterobacter*, 9 (4.4%) *Escherichia*, etc.

The MALDI-TOF MS results of the Autof ms1000 and Bruker Biotyper are summarized in Table 1. The Autof ms1000 achieved species-level identification for 202 (98.5%) strains, genus-level identification for 1 (0.5%) strain, and no reliable results for 2 (1.0%) strains. The Bruker Biotyper achieved species-level identification for 200 (97.6%) strains and genus-level identification for 5 (2.4%) strains. For the Autof ms1000, the 3 nonspecies results were 1 (8.3%, genus identification) strain of anaerobes and 2 (9.1%, no reliable identification) strains of fungi. For the Bruker Biotyper, the 5 nonspecies results were 2 (2.6%, genus identification) strains in Gram-positive cocci and 3 (13.6%, genus identification) strains in fungi. The data of preserved strains is presented in File S1 of the Supplementary Material (available here).

### 3.2. Comparison of Identification Performance in Preserved Strains

Comparing the overall identification performance, the Autof ms1000 correctly identified 200 strains at the species/complex level and 1 strain at the genus level (Table 2). The Bruker Biotyper correctly identified 199 strains at the species/complex level and 5 strains at the genus level. There was no significant difference in the species/complex level identification performance of the two instruments (*P* value = 1.000). The Autof ms1000 and Bruker Biotyper misidentified 2 strains and 1 strain, respectively. The correct results of the Autof ms1000 were Enterobacterales 98.3% (58/59), nonfermenters 96.2% (25/26), other Gram-negative rods 100.0% (6/6), Gram-positive cocci 100.0% (77/77), Gram-positive rods 100.0% (3/3), anaerobes 91.7% (11/12), and fungi 90.9% (20/22). The correct results of the Bruker Biotyper were Enterobacterales 98.3% (58/59), nonfermenters 96.2% (25/26), other Gram-negative rods 100.0% (6/6), Gram-positive cocci 97.4% (75/77), Gram-positive rods 100.0% (3/3), anaerobes 100.0% (12/12), and fungi 86.4% (19/22).

### 3.3. Identification Results in Clinical Isolates

A total of 302 clinical isolates were used for evaluation, of which 174 were Gram-negatives, 118 Gram-positives, and 10 fungi. Their genera were *Staphylococcus* (59, 19.5%), *Escherichia* (54, 17.9%), *Enterococcus* (49, 16.2%), *Klebsiella* (31, 10.3%), *Enterobacter* (24, 8.0%), *Pseudomonas* (24, 8.0%), *Streptococcus* (10, 3.3%), etc. The MALDI-TOF MS results of both instruments are presented in Table 3. The results of the Autof ms1000 were 301 (99.7%) species-level identification and 1 (0.3%) no reliable result. The results of the Bruker Biotyper were 300 (99.3%) species-level identification and 2 (0.7%) genus-level identification. No species-level results were obtained in 1 (10.0%) yeast with no reliable results by the Autof ms1000 and 2 (1.7%) Gram-positive cocci with genus-level results by the Bruker Biotyper. The data of clinical isolates is presented in File S1 of the Supplementary Material.

### 3.4. Comparison of Identification Performance in Clinical Isolates

In the comparison of the overall performance, the correct results obtained by the Autof ms1000 were 299 isolates at the species/complex level, and the correct results obtained by the Bruker Biotyper were 300 isolates at the species/complex level and 2 isolates at the genus level (Table 4). There was no significant difference in the species/complex-level identification performance of the two instruments (*P* value = 1.000). The Autof ms1000 produced 1 case with no reliable result and 2 misidentifications. There were no unreliable results or misidentifications by the Bruker Biotyper. The correct results of the Autof ms1000 were Enterobacterales 99.2% (130/131), nonfermenters 100.0% (41/41), other Gram-negative rods 100.0% (2/2), Gram-positive cocci 99.2% (117/118), and fungi 90.0% (9/10). The correct results of the Bruker Biotyper were Enterobacterales 100.0% (131/131), nonfermenters 100.0% (41/41), other Gram-negative rods 100.0% (2/2), Gram-positive cocci 98.4% (116/118), and fungi 100.0% (10/10).

### 3.5. Inconsistent Results between the Autof ms1000 and the Bruker Biotyper

Discrepancies between the species-complex results obtained by the two instruments are summarized in Table 5. There were 5 misidentifications produced by the Autof ms1000 and 1 by the Bruker Biotyper. The strain incorrectly identified by both instruments was *S. nematodiphila*, which was misidentified as *S. marcescens* by the Autof ms1000 and *S. ureilytica* by the Bruker Biotyper. *E. gallinarum* was misidentified as *E. casseliflavus* for the Autof ms1000, and the Bruker Biotyper produced the same result but at the genus level. In addition, the Autof ms1000 incorrectly identified *A. bereziniae* as *A. guillouiae* and *K. oxytoca* as *R. ornithinolytica*. Due to the low range/score values, there were 3 cases of no reliable results for the Autof ms1000, all of which were *Candida auris*. The Bruker Biotyper identified 2 strains of *C. auris* at the species level and 1 at the genus level. The genus-level results were 1 strain with the Autof ms1000 and 7 strains with the Bruker Biotyper. Except for *E. casseliflavus*, the genus-level results were consistent with the species results.

### 3.6. Comparison of the Analysis Time

The target plate was placed on the device, and then, the test preparation time and the time required to analyze 12 and 96 spots were measured (Table 6). Loading the target plate and establishing vacuum took 36 sec for the Autof ms1000 and 1 min 33 sec for the Bruker Biotyper (*P* value < 0.001). When twelve spots were analyzed, the test times for the Autof ms1000 and Bruker Biotyper were 1 min 40 sec and 3 min 13 sec, respectively (*P* value < 0.001). In addition, the Autof ms1000 took 13 min 52 sec to analyze 96 spots, and the Bruker Biotyper took 25 min 38 sec.

## 4. Discussion

This study showed that the species-level identification performance of the Autof ms1000 and Bruker Biotyper in clinically significant strains is comparable at 97% or more. Considering microorganisms that are difficult to identify with MALDI-TOF MS, excellent agreement was confirmed. These results used conventional extraction methods and may be applicable to practical use in clinical microbiology laboratories.

Results were obtained at the genus level for 7 isolates by the Bruker Biotyper but for 1 isolate by the Autof ms1000. Five of these results were for Gram-positive cocci and 2 for fungi. In a comparative evaluation with the VITEK MS, the Bruker Biotyper achieved lower species-level identification rates in Gram-positive and yeast strains than the VITEK MS [6, 8]. However, when genus-level results were interpreted as species-level identification, the results were mostly consistent, and similar results were obtained (85.7%, 6/7 isolates).

The Autof ms1000 provided species-level results that were actual misidentifications in 4 isolates, whereas the Bruker Biotyper provided 1. *S. marcecens* and *S. ureilytica* are known to be difficult to distinguish by MALDI-TOF MS, and *S. nematodiphila* is a new strain reported in 2009 and seems to be misidentified in both instruments because it is phylogenetically close to other strains [25]. The Autof ms1000 incorrectly identified *A. bereziniae* as *A. guillouiae*. Both bacteria were proposed together as novel species in 2010 and known to be phylogenetically close but can be distinguished by specific signals in MALDI-TOF MS [26]. The misidentification may have occurred because *A. bereziniae* is not included in the Autof ms1000 database. Both devices identified *E. gallinarum* as *E. casseliflavus*, and the results were species level in the Autof ms1000 and genus level in the Bruker Biotyper. It has been reported that MALDI-TOF MS can accurately identify *Enterococcus* at the species level [27]. The *E. gallinarum* strain was considered a challenging strain to identify by MALDI-TOF MS, since both devices showed correct results for two *E. casseliflavus* strains. *K. oxytoca* was identified as *R. ornithinolytica* in the Autof ms1000, and these species are also known to be difficult to distinguish by MALDI-TOF MS [28].

Autof ms1000 failed to identify all three strains of *C. auris*. *C. auris* is an emerging pathogen in infection prevention due to reported nosocomial outbreaks and high rates of antifungal resistance [29]. In 2019, the US Centers for Disease Control and Prevention (CDC) recommended the use of MALDI-TOF MS for the identification of *C. auris* and announced available instruments and database version information. It was confirmed that *C. auris* was not included in the installed database of Autof ms1000 used in this evaluation. The manufacturer recently updated the cloud database with *C. auris*, and the spectra obtained by EtOH/FA extraction were analyzed with the cloud database and correctly identified (range: 9.271-9.521).

Among the misidentifications, it was the most clinically important that the Autof ms1000 failed to identify *C. auris*. As a result, appropriate measures such as infection control and antifungal prescription may be delayed [29]. Additionally, the Autof ms1000 incorrectly identified *K. oxytoca* as *R. ornithinolyticus*, a rare pathogen in human infection [30]. With the development of identification technology, recent reports on the pathogenicity of *R. ornithinolyticus* have been made [30]. Other species-level misidentifications, including *Serratia* spp., *Acinetobacter* spp., and *Enterococcus* spp., had little clinical significance, as they did not alter the interpretation of antimicrobial susceptibility [31]. An important misidentification confirmed in the recent Autof ms1000 evaluation in China is that the Bruker Biotyper failed to identify *B. pseudomallei* because it was not included in the database [17].

The Autof ms1000 has the advantage of using the latest library free of charge through the cloud database. The Bruker Biotyper does not support cloud databases, and the cost of updating the database is a burden for the laboratory. In this evaluation, we used an installed database created in June 2019 because the cloud database was unavailable due to the hospital's security policy. The real analysis of the Autof ms1000 using the latest database is performed in the cloud, and the database is not downloadable. However, as medical institutions' network security is becoming increasingly important [32], Internet access may not be possible. Therefore, it is necessary to update the MALDI-TOF MS database regularly. Unlike the Bruker Biotyper, the Autof ms1000 has the disadvantage that users cannot check the species included in the database with software. Since the role of MALDI-TOF MS has been emphasized in recent cutaneous diphtheria cases [33], it should be possible to check the database when there is a related issue.

The Autof ms1000 and Bruker Biotyper have similar ease of use. Both MALDI-TOF MS systems are benchtops, but the Autof ms1000 has an external vacuum pump, which makes more noise. Neither device provides a ready-to-use matrix solution, and the matrix solution and the calibrator are provided lyophilized. In addition, 70% FA and ACN are used in the extraction steps for both instruments. The Autof ms1000 provides only disposable target plates, whereas the Bruker Biotyper provides both reusable and disposable target plates.

When comparing the time for identification, the Autof ms1000 was faster than the Bruker Biotyper in both the time for loading the target plate and achieving vacuum and the time for analyzing the plate. These times were reported as 2-3 min and 40-50 min (96 spots) for the Bruker Biotyper and as 5-6 min and 45-55 min (96 spots) for the VITEK MS [3, 8]. The time taken for the examination did not differ from that in the reports. The difference in time for loading the target plate and achieving vacuum seems to be due to an external pump of the Autof ms1000. Additionally, the Autof ms1000 optimized the order of reading spots and reduced moving time. For example, the Bruker Biotyper tests the A12 spot and then the B1 spot, while the Autof ms1000 tests the A12 spot and then the B12 spot. Considering using 10-20 spots at a time with MALDI-TOF MS in a clinical microbiology laboratory, the analysis time of the Autof ms1000 is more than twice as fast as that of the Bruker Biotyper, making it convenient for laboratories. However, the impact of rapid MALDI-TOF MS on actual reporting time and workflow in laboratories should be investigated in further studies.

## 5. Conclusions

The Autof ms1000 showed comparable identification performance to that of the Bruker Biotyper. The Autof ms1000 provided more species-level results in Gram-positive cocci and fungi than the Bruker Biotyper but produced more misidentifications due to a database problem. In particular, the test time of the Autof ms1000 was approximately half that of the Bruker Biotyper, which is helpful in providing higher throughput in the clinical microbiology laboratory.

## Figures and Tables

**Table 1:** Identification results of preserved strains.

Group of organisms	*N*	Autof ms1000			Bruker Biotyper		
		Species (%)	Genus (%)	No reliable ID (%)	Species (%)	Genus (%)	No reliable ID (%)
Enterobacterales	59	59 (100.0)	0 (0.0)	0 (0.0)	59 (100.0)	0 (0.0)	0 (0.0)
Nonfermenters	26	26 (100.0)	0 (0.0)	0 (0.0)	26 (100.0)	0 (0.0)	0 (0.0)
Other Gram-negative rods	6	6 (100.0)	0 (0.0)	0 (0.0)	6 (100.0)	0 (0.0)	0 (0.0)
Gram-positive cocci	77	77 (100.0)	0 (0.0)	0 (0.0)	75 (97.4)	2 (2.6)	0 (0.0)
Gram-positive rods	3	3 (100.0)	0 (0.0)	0 (0.0)	3 (100.0)	0 (0.0)	0 (0.0)
Anaerobes	12	11 (91.7)	1 (8.3)	0 (0.0)	12 (100.0)	0 (0.0)	0 (0.0)
Fungi	22	20 (90.9)	0 (0.0)	2 (9.1)	19 (86.4)	3 (13.6)	0 (0.0)
Total	205	202 (98.5)	1 (0.5)	2 (1.0)	200 (97.6)	5 (2.4)	0 (0.0)

Abbreviation: ID = identification.

**Table 2:** Comparison of identification performance in preserved strains.

Species	*N*	Autof ms1000				Bruker Biotyper			
		ID species	ID genus	No ID	MisID species	ID species	ID genus	No ID	MisID species
Enterobacterales (%)	59	58 (98.3)	0 (0.0)	0 (0.0)	1 (1.7)	58 (98.3)	0 (0.0)	0 (0.0)	1 (1.7)
*Citrobacter freundii* complex	6	6	0	0	0	6	0	0	0
*Enterobacter aerogenes*	3	3	0	0	0	3	0	0	0
*Enterobacter cloacae* complex	8	8	0	0	0	8	0	0	0
*Escherichia coli*	9	9	0	0	0	9	0	0	0
*Hafnia alvei*	1	1	0	0	0	1	0	0	0
*Klebsiella oxytoca*	3	3	0	0	0	3	0	0	0
*Klebsiella pneumoniae* complex	9	9	0	0	0	9	0	0	0
*Morganella morganii*	3	3	0	0	0	3	0	0	0
*Pantoea agglomerans*	1	1	0	0	0	1	0	0	0
*Proteus mirabilis*	5	5	0	0	0	5	0	0	0
*Proteus vulgaris*	2	2	0	0	0	2	0	0	0
*Providencia rettgeri*	1	1	0	0	0	1	0	0	0
*Providencia stuartii*	1	1	0	0	0	1	0	0	0
*Salmonella* species	2	2	0	0	0	2	0	0	0
*Serratia liquefaciens*	1	1	0	0	0	1	0	0	0
*Serratia marcescens*	2	2	0	0	0	2	0	0	0
*Serratia nematodiphila*	1	0	0	0	1	0	0	0	1
*Yersinia enterocolitica*	1	1	0	0	0	1	0	0	0
Nonfermenters (%)	26	25 (96.2)	0 (0.0)	0 (0.0)	1 (3.8)	26 (100.0)	0 (0.0)	0 (0.0)	0 (0.0)
*Achromobacter xylosoxidans*	2	2	0	0	0	2	0	0	0
*Acinetobacter baumannii* complex	6	6	0	0	0	6	0	0	0
*Acinetobacter bereziniae*	1	0	0	0	1	1	0	0	0
*Burkholderia cepacia* complex	2	2	0	0	0	2	0	0	0
*Moraxella catarrhalis*	2	2	0	0	0	2	0	0	0
*Pseudomonas aeruginosa*	8	8	0	0	0	8	0	0	0
*Pseudomonas stutzeri*	1	1	0	0	0	1	0	0	0
*Stenotrophomonas maltophilia*	4	4	0	0	0	4	0	0	0
Other Gram-negative rods (%)	6	6 (100.0)	0 (0.0)	0 (0.0)	0 (0.0)	6 (100.0)	0 (0.0)	0 (0.0)	0 (0.0)
*Campylobacter jejuni*	1	1	0	0	0	1	0	0	0
*Haemophilus influenzae*	3	3	0	0	0	3	0	0	0
*Haemophilus parainfluenzae*	1	1	0	0	0	1	0	0	0
*Neisseria gonorrhoeae*	1	1	0	0	0	1	0	0	0
Gram-positive cocci (%)	77	77 (100.0)	0 (0.0)	0 (0.0)	0 (0.0)	75 (97.4)	2 (2.6)	0 (0.0)	0 (0.0)
*Aerococcus viridans*	1	1	0	0	0	1	0	0	0
*Enterococcus avium*	1	1	0	0	0	1	0	0	0
*Enterococcus casseliflavus*	2	2	0	0	0	2	0	0	0
*Enterococcus faecalis*	8	8	0	0	0	8	0	0	0
*Enterococcus faecium*	9	9	0	0	0	9	0	0	0
*Enterococcus raffinosus*	1	1	0	0	0	1	0	0	0
*Micrococcus luteus*	1	1	0	0	0	1	0	0	0
*Parvimonas micra*	1	1	0	0	0	1	0	0	0
*Rothia mucilaginosa*	1	1	0	0	0	1	0	0	0
*Staphylococcus aureus*	10	10	0	0	0	10	0	0	0
*Staphylococcus capitis*	2	2	0	0	0	2	0	0	0
*Staphylococcus epidermidis*	9	9	0	0	0	9	0	0	0
*Staphylococcus haemolyticus*	3	3	0	0	0	3	0	0	0
*Staphylococcus hominis*	1	1	0	0	0	1	0	0	0
*Staphylococcus lugdunensis*	1	1	0	0	0	1	0	0	0
*Staphylococcus pasteuri*	1	1	0	0	0	1	0	0	0
*Staphylococcus saprophyticus*	2	2	0	0	0	2	0	0	0
*Streptococcus agalactiae*	6	6	0	0	0	6	0	0	0
*Streptococcus anginosus*	3	3	0	0	0	3	0	0	0
*Streptococcus constellatus*	2	2	0	0	0	2	0	0	0
*Streptococcus dysgalactiae*	1	1	0	0	0	1	0	0	0
*Streptococcus gallolyticus*	1	1	0	0	0	1	0	0	0
*Streptococcus intermedius*	1	1	0	0	0	1	0	0	0
*Streptococcus oralis*	1	1	0	0	0	1	0	0	0
*Streptococcus parasanguinis*	1	1	0	0	0	0	1	0	0
*Streptococcus pneumoniae*	2	2	0	0	0	1	1	0	0
*Streptococcus pyogenes*	3	3	0	0	0	3	0	0	0
*Streptococcus salivarius*	2	2	0	0	0	2	0	0	0
Gram-positive rods (%)	3	3 (100.0)	0 (0.0)	0 (0.0)	0 (0.0)	3 (100.0)	0 (0.0)	0 (0.0)	0 (0.0)
*Corynebacterium striatum*	2	2	0	0	0	2	0	0	0
*Listeria monocytogenes*	1	1	0	0	0	1	0	0	0
Anaerobes (%)	12	11 (91.7)	1 (8.3)	0 (0.0)	0 (0.0)	12 (100.0)	0 (0.0)	0 (0.0)	0 (0.0)
*Bacteroides fragilis*	2	2	0	0	0	2	0	0	0
*Bacteroides ovatus*	1	1	0	0	0	1	0	0	0
*Bacteroides thetaiotaomicron*	1	1	0	0	0	1	0	0	0
*Clostridioides difficile*	1	1	0	0	0	1	0	0	0
*Clostridium perfringens*	1	1	0	0	0	1	0	0	0
*Eggerthella lenta*	1	0	1	0	0	1	0	0	0
*Fusobacterium nucleatum*	1	1	0	0	0	1	0	0	0
*Prevotella melaninogenica*	1	1	0	0	0	1	0	0	0
*Propionibacterium acnes*	2	2	0	0	0	2	0	0	0
*Veillonella parvula*	1	1	0	0	0	1	0	0	0
Fungi (%)	22	20 (90.9)	0 (0.0)	2 (9.1)	0 (0.0)	19 (86.4)	3 (13.6)	0 (0.0)	0 (0.0)
*Candida albicans*	5	5	0	0	0	5	0	0	0
*Candida auris*	2	0	0	2	0	1	1	0	0
*Candida dubliniensis*	1	1	0	0	0	1	0	0	0
*Candida glabrata*	3	3	0	0	0	2	1	0	0
*Candida krusei*	2	2	0	0	0	2	0	0	0
*Candida orthopsilosis*	1	1	0	0	0	0	1	0	0
*Candida parapsilosis*	3	3	0	0	0	3	0	0	0
*Candida tropicalis*	4	4	0	0	0	4	0	0	0
*Cryptococcus neoformans*	1	1	0	0	0	1	0	0	0
Total (%)	205	200 (97.6)	1 (0.5)	2 (1.0)	2 (1.0)	199 (97.1)	5 (2.4)	0 (0.0)	1 (0.5)

Abbreviations: ID = identification; MisID = misidentification.

**Table 3:** Identification results in clinical isolates.

Group of organisms	*N*	Autof ms1000			Bruker Biotyper		
		Species (%)	Genus (%)	No reliable ID (%)	Species (%)	Genus (%)	No reliable ID (%)
Enterobacterales	131	131 (100.0)	0 (0.0)	0 (0.0)	131 (100.0)	0 (0.0)	0 (0.0)
Nonfermenters	41	41 (100.0)	0 (0.0)	0 (0.0)	41 (100.0)	0 (0.0)	0 (0.0)
Other Gram-negative rods	2	2 (100.0)	0 (0.0)	0 (0.0)	2 (100.0)	0 (0.0)	0 (0.0)
Gram-positive cocci	118	118 (100.0)	0 (0.0)	0 (0.0)	116 (98.3)	2 (1.7)	0 (0.0)
Fungi	10	9 (90.0)	0 (0.0)	1 (10.0)	10 (100.0)	0 (0.0)	0 (0.0)
Total	302	301 (99.7)	0 (0.0)	1 (0.3)	300 (99.3)	2 (0.7)	0 (0.0)

Abbreviation: ID = identification.

**Table 4:** Comparison of identification performance in clinical isolates.

Species	*N*	Autof ms1000				Bruker Biotyper			
		ID species	ID genus	No ID	MisID species	ID species	ID genus	No ID	MisID species
Enterobacterales (%)	131	130 (99.2)	0 (0.0)	0 (0.0)	1 (0.8)	131 (100.0)	0 (0.0)	0 (0.0)	0 (0.0)
*Citrobacter freundii* complex	6	6	0	0	0	6	0	0	0
*Enterobacter aerogenes*	10	10	0	0	0	10	0	0	0
*Enterobacter cloacae* complex	14	14	0	0	0	14	0	0	0
*Escherichia coli*	54	54	0	0	0	54	0	0	0
*Klebsiella oxytoca*	2	1	0	0	1	2	0	0	0
*Klebsiella pneumoniae* complex	29	29	0	0	0	29	0	0	0
*Morganella morganii*	3	3	0	0	0	3	0	0	0
*Proteus mirabilis*	6	6	0	0	0	6	0	0	0
*Raoultella ornithinolytica*	4	4	0	0	0	4	0	0	0
*Serratia marcescens*	3	3	0	0	0	3	0	0	0
Nonfermenters (%)	41	41 (100.0)	0 (0.0)	0 (0.0)	0 (0.0)	41 (100.0)	0 (0.0)	0 (0.0)	0 (0.0)
*Acinetobacter baumannii* complex	9	9	0	0	0	9	0	0	0
*Elizabethkingia meningoseptica*	1	1	0	0	0	1	0	0	0
*Pseudomonas aeruginosa*	24	24	0	0	0	24	0	0	0
*Stenotrophomonas maltophilia*	7	7	0	0	0	7	0	0	0
Other Gram-negative rods (%)	2	2 (100.0)	0 (0.0)	0 (0.0)	0 (0.0)	2 (100.0)	0 (0.0)	0 (0.0)	0 (0.0)
*Haemophilus influenzae*	2	2	0	0	0	2	0	0	0
Gram-positive cocci (%)	118	117 (99.2)	0 (0.0)	0 (0.0)	1 (0.8)	116 (98.3)	2 (1.7)	0 (0.0)	0 (0.0)
*Enterococcus avium*	1	1	0	0	0	1	0	0	0
*Enterococcus faecalis*	15	15	0	0	0	15	0	0	0
*Enterococcus faecium*	32	32	0	0	0	32	0	0	0
*Enterococcus gallinarum*	1	0	0	0	1	0	1	0	0
*Staphylococcus aureus*	29	29	0	0	0	29	0	0	0
*Staphylococcus capitis*	3	3	0	0	0	3	0	0	0
*Staphylococcus caprae*	1	1	0	0	0	1	0	0	0
*Staphylococcus epidermidis*	16	16	0	0	0	15	1	0	0
*Staphylococcus haemolyticus*	2	2	0	0	0	2	0	0	0
*Staphylococcus hominis*	5	5	0	0	0	5	0	0	0
*Staphylococcus lugdunensis*	3	3	0	0	0	3	0	0	0
*Streptococcus agalactiae*	8	8	0	0	0	8	0	0	0
*Streptococcus anginosus*	1	1	0	0	0	1	0	0	0
*Streptococcus pyogenes*	1	1	0	0	0	1	0	0	0
Fungi (%)	10	9 (90.0)	0 (0.0)	1 (10.0)	0 (0.0)	10 (100.0)	0 (0.0)	0 (0.0)	0 (0.0)
*Candida albicans*	4	4	0	0	0	4	0	0	0
*Candida auris*	1	0	0	1	0	1	0	0	0
*Candida krusei*	3	3	0	0	0	3	0	0	0
*Candida lusitaniae*	1	1	0	0	0	1	0	0	0
*Rhodotorula mucilaginosa*	1	1	0	0	0	1	0	0	0
Total (%)	302	299 (99.0)	0 (0.0)	1 (0.3)	2 (0.7)	300 (99.3)	2 (0.7)	0 (0.0)	0 (0.0)

Abbreviations: ID = identification; MisID = misidentification; NA = not applicable.

**Table 5:** Detailed results of microorganisms that either were misidentified or could not be identified by one or both MALDI-TOF MS systems.

Test groups	Reference	Autof ms1000			Bruker Biotyper		
		Result	Range	Agreement	Result	Score	Agreement
Preserved strains	*Streptococcus pneumoniae*	*S. pneumoniae*	9.400	ID species	*S. pneumoniae*	1.924	ID genus
	*Serratia nematodiphila*	*S. marcescens*	9.431	MisID species	*S. ureilytica*	2.136	MisID species
	*Candida orthopsilosis*	*C. orthopsilosis*	9.507	ID species	*C. orthopsilosis*	1.82	ID genus
	*Streptococcus parasanguinis*	*S. parasanguinis*	9.361	ID species	*S. parasanguinis*	1.907	ID genus
	*Candida glabrata*	*C. glabrata*	9.441	ID species	*C. glabrata*	1.866	ID genus
	*Eggerthella lenta*	*E. lenta*	8.967	ID genus	*E. lenta*	2.114	ID species
	*Acinetobacter bereziniae*	*A. guillouiae*	9.127	MisID species	*A. bereziniae*	2.177	ID species
	*Candida auris*	No reliable ID	5.401	No ID	*C. auris*	2.031	ID species
	*Candida auris*	No reliable ID	4.755	No ID	*C. auris*	1.892	ID genus
Clinical isolates	*Candida auris*	No reliable ID	4.952	No ID	*C. auris*	2.073	ID species
	*Enterococcus gallinarum*	*E. casseliflavus*	9.543	MisID species	*E. casseliflavus*	1.905	ID genus
	*Klebsiella oxytoca*	*Raoultella ornithinolytica*	9.159	MisID species	*K. oxytoca*	2.173	ID species
	*Staphylococcus epidermidis*	*S. epidermidis*	9.508	ID species	*S. epidermidis*	1.982	ID genus

Abbreviations: ID = identification; MisID = misidentification.

**Table 6:** Comparison of time to identification by Autof ms1000 and Bruker Biotyper.

Description	Autof ms1000	Bruker Biotyper	*P* value
Time to load target plate and make vacuum (*n* = 14)	36 sec (35 sec-37 sec)	1 min 33 sec (1 min 26 sec-1 min 53 sec)	<0.001
Time to analyze 12 spots (*n* = 37)	1 min 40 sec (1 min 31 sec-1 min 52 sec)	3 min 13 sec (2 min 58 sec-3 min 45 sec)	<0.001
Time to analyze 96 spots (*n* = 3)	13 min 52 sec (13 min 35 sec-13 min 55 sec)	25 min 38 sec (22 min 17 sec-27 min 56 sec)	NA

Abbreviation: NA = not applicable.

## Data Availability

The data used to support the findings of this study are included within the supplementary information file.

## Supplementary Materials

Supplementary Materials Supplementary File 1: the MALDI-TOF MS results in preserved strains and clinical isolates.
